# Parenting Style and Emotional Distress Among Chinese College Students: A Potential Mediating Role of the Zhongyong Thinking Style

**DOI:** 10.3389/fpsyg.2020.01774

**Published:** 2020-07-28

**Authors:** Yanfei Hou, Rong Xiao, Xueling Yang, Yu Chen, Fei Peng, Shegang Zhou, Xihua Zeng, Xiaoyuan Zhang

**Affiliations:** ^1^Department of Psychology, School of Public Health, Southern Medical University, Guangzhou, China; ^2^School of Nursing, Southern Medical University, Guangzhou, China

**Keywords:** zhongyong thinking, parenting style, depressive symptom, anxiety symptom, mediating and moderating effect

## Abstract

Previous studies suggested that parenting style was associated with college student’s emotional distress. However, little is known about the underlying mechanisms of this relation in Chinese culture. The present study investigated the associations between parenting style and college student’s emotional distress (depression and anxiety symptoms), examined the mediating effects of Confucian personality-Zhongyong thinking, and explored whether gender, age, and socioeconomic status (SES) moderated the direct and/or indirect effects of parenting style on emotional distress. Results from a large representative sample of Chinese college students (*n* = 3943) indicated that (a) parental rejection and overprotection was positively and mildly associated with depressive and anxiety symptoms and negatively and mildly related to Zhongyong thinking. Parental warmth significantly correlated with the three variables in the opposite direction; Zhongyong thinking correlated negatively and moderately with depression, and mildly with anxiety; (b) Zhongyong thinking partially mediated the associations of parental rejection and warmth with emotional distress. Specifically, to the extent that students perceived less rejection and more warmth, they were more likely to develop Zhongyong thinking associated with decreased emotional distress; (c) gender and SES moderated the association between parenting style and Zhongyong thinking. Specifically, for students with low SES, the negative relationship between parental overprotection and Zhongyong thinking was stronger; for males and high SES students, the positive link between parental warmth and Zhongyong thinking were stronger. Results highlight the importance of researching potential effects of college student’s Zhongyong thinking within the family system in Chinese culture.

## Introduction

Depressive and anxiety symptoms are among the most important public health issues globally, and these common mental health problems increasingly affect college students (Patel et al., 2007; Auerbach et al., 2018). According to the report of the WHO World Mental Health International College Student project (*n* = 13,984), the estimated prevalence of depressive and anxiety symptoms was 21.2 and 16.7%, respectively (Auerbach et al., 2018). A meta-analysis of 32,694 Chinese college students indicated that the prevalence of depressive symptoms among them was 23.8% (Lei et al., 2016). These symptoms interfere with the way they think, feel, and act; limit their academic achievements and ability to be economically productive; and lead to psychological disorders and sometimes suicide (Nguyen et al., 2013). Therefore, the potential factors that contribute to college student’s emotional distress and the mechanisms urgently need to be studied.

### Parenting Style and Emotional Distress

Among the factors affecting the depression and anxiety symptoms of college students, the role of the parents has received great attention, and parenting style is one of the most frequently studied parental dimensions. For college students, the education period is prolonged and the transition to self-sufficient adulthood is delayed. Parents continue to play a vital role in college student’s lives, such as providing financial and emotional support for them (Guan and Fuligni, 2016). Therefore, these young adult college students may be still greatly influenced by their parents and the parenting styles.

Parenting style is conceptualized as a constellation of attitudes toward the offspring that are communicated to them and the emotional climate in which parent’s attitudes are expressed (Darling and Steinberg, 1993). Parenting styles usually falls on the continuum between the two anchors of laxity and excessive punishment, and extremes in either direction are defined as negative (Stevens, 2014; Xu et al., 2017). Positive parenting style is characterized by a high level of perceived parental warmth (e.g., support and acceptance); in contrast, negative parenting style is characterized by parental rejection and overprotection (e.g., harsh parenting, coercive or authoritarian interactions, and strict regulation and monitoring) (Vera et al., 2012; Lian et al., 2016). Research on parenting styles has repeatedly shown that parenting style may have an important and long-term impact on emotional distress among the offspring (Ebrahimi et al., 2017). Specifically, a recalled negative parenting style is associated with adult’s high levels of depression and anxiety, while a positive parenting style is linked with low levels of depression and anxiety among them (Rodriguez et al., 2016; Ernst et al., 2020). For instance, Ebrahimi et al. (2017) used a sample of graduate students and found a negative relationship between authoritative parenting style (high acceptance and support) and student’s depression and a positive relationship between authoritarian parenting style (high control and low support) and student’s depression. A survey on emerging adult college students in America indicated that perceived authoritative parenting style was associated with their fewer anxiety symptoms (Rodriguez et al., 2016). Furthermore, in a sample of German adult childhood-cancer survivors, recalled parenting style was related to their lifetime diagnoses of depression and anxiety disorders (Ernst et al., 2020). These findings highlight that the relationship between parenting style and adult emotional distress risks has been well established.

However, most of the above studies were conducted in Western countries. Although it has been proved that cultural factors impact on patterns of the association between parenting style and individual’s emotion (Pinquart and Kauser, 2018), there is still a few literature focusing on the relationships between parenting style and adult’s emotional distress in Chinese culture. Li et al. (2010) surveyed a small sample of Chinese college studnets (*N* = 79) and found that maternal authoritarian parenting style was negatively associated with student’s depression. Then, Gao et al. (2012) found that parental warmth was related to a low risk for major depression. However, the participants were all adult women. Therefore, the link between parenting style and emotion among Chinese adult men was unknown. In 2016, a survey on 439 Chinese elders aged 60–91 years showed that the elders who recalled a high frequency of authoritarian parenting style had high levels of depression and anxiety (Zhong et al., 2016). Similarly, Cui (2017) surveyed a sample of 545 college students in China and reported that perceived high overprotection and low warmth correlated with student’s high levels of depression and anxiety symptoms. These results highlighted that parenting style perceived by Chinese adults would be closely associated with their depression and anxiety symptoms. Recently, research has began to explore the underlying mechanisms underlying these relations. For example, a recent study (Hong and Cui, 2020) found that the links between helicopter parenting (high warmth and high control) and depression and anxiety symptoms were mediated by college student’s self-control. Such research efforts, however, remain scarce. Much less is known about other potential mechanisms.

### Zhongyong Thinking and Emotional Distress

Zhongyong thinking (Doctrine of the Mean, Middle-way thinking), as the central theme of Confucian personality, is the most influential thinking style in China (Chang and Yang, 2014). It is also the recommended mode of action to be applied to interpersonal interactions among Chinese individuals (Yao et al., 2010). Based on the theory of Wu and Lin (2005), Zhongyong thinking is defined as thinking about things carefully from various aspects and taking appropriate actions to account for the whole situation, and it comprises three features: multiple thinking, holism, and harmoniousness. Specifically, multivariate thinking requires individuals to think from multiple perspectives in the context of expressing opinions, that is, to make decisions after considering various possibilities from multiple perspectives. Holism measures the degree to which external information and internal requirements are integrated as a whole. Harmoniousness assesses the tendency to act in harmony when handling interpersonal conflicts. Essentially, individuals with high Zhongyong thinking would avoid going to extremes and show appropriate behaviors under different situations, according to the specific needs of situational context and intrinsic personal expectations (Peng and Nisbett, 1999; Zhou et al., 2019). Furthermore, maintaining interpersonal harmony is the ultimate goal of Zhongyong thinking (Wu and Lin, 2005; Chen et al., 2015). Zhongyong thinking plays an important role as Chinese college students head for a progressively independent and challenging life. Specifically, in order to effectively manage their academic challenges, interpersonal relationships, finances, and health issues, college students need to substantially rely on Zhongyong thinking to avoid contradictions and actively coordinating conflicts. Failure to fulfill these social adaptive goals and maintain harmony would have negative impacts on their emotion.

The diathesis-stress model could help explain why Zhongyong thinking could be related to college student’s emotion. Based on the diathesis-stress model, individuals with some personality traits, such as poor self-esteem (Nguyen et al., 2019), low self-directedness and cooperativeness (Lim et al., 2018), low agreeableness (Roman et al., 2019), and high perfectionism (Bußenius and Harendza, 2019), may predispose them to develop more depression and anxiety symptoms. Results from two cross-sectional studies demonstrated that Chinese adults with high level of Zhongyong thinking style had few depressive and anxiety symptoms (Zhan et al., 2013; Yang et al., 2016). Moreover, Yang et al. (2016) conducted a longitudinal study and found that Chinese college student’s depressive symptoms could be relieved through the training of Zhongyong thinking in group psychotherapy. Therefore, in China, the Zhongyong thinking may be an important protective personality trait for an individual’s emotional distress, and Zhongyong thinking is considered to be negatively related to Chinese individual’s emotional distress. However, little work has examined the link of Zhongyong thinking with emotional distress when considering the influence of parenting style.

### The Mediating Role of Zhongyong Thinking

The research of the mechanism of parenting style on college student’s emotional distress is essential to better understand the theory in this field and to develop effective interventions to prevent or decrease emotional distress for college students. According to the ecological system model, parenting style is a crucial family factor affecting the personality development of the offspring (Heaven and Ciarrochi, 2008; Pérez-Fuentes et al., 2019). For example, Heaven and Ciarrochi (2008) conducted a longitudinal study and found that the authoritative parenting style that students perceived in seventh grade predicted an increase in student’s conscientiousness in eighth grade. Furthermore, Hong and Cui (2020) found in Chinese college students that, self-control, the ability to voluntarily resist immediate rewards or suppress undesirable impulses that conflict with long-term goals, served as a mediating mechanism through which helicopter parenting was linked to college student’s depressive and anxiety symptoms. However, the existing research only provided limited indirect evidence of the associations between parenting style and Zhongyong thinking. Specifically, a cross-sectional study of a sample of teenagers found that family function had a significantly positive relationship with the level of the individual’s Zhongyong thinking (Yang, 2012), and parenting style was the main determinant of family functioning (Matejevic et al., 2014). Based on the above theoretical work and empirical studies, it is reasonable to infer that Zhongyong thinking style, an adaptive personality trait in Chinese culture, may also mediate the association between perceived parenting style and college student’s emotional distress.

### The Moderating Role of Age, Gender, and Socioeconomic Status (SES)

In current study, we test gender, age, and SES as potential moderators. Research of these issues would not only benefit the understanding of gender-related, age-related, and/or SES related patterns in the association between parenting style and emotional distress, but also has direct contribution for gender-specific, age-specific, and/or SES specific emotion-related interventions.

There are several reasons for examining for gender differences in the association between parenting style and emotional distress among college students. Research has shown that females tend to have higher prevalence of emotional distress than males (McLean and Anderson, 2009). Furthermore, females are more influenced by relationships (Cyranowski et al., 2000) and parenting style (Barton and Kirtley, 2012). For instance, in one study of college students, helicopter parenting was associated with lower levels of well-being for females only (Kouros et al., 2017). However, to our knowledge, no studies have tested the effect of gender on the link between parenting style and Zhongyong thinking, or between Zhongyong thinking and emotional distress in college students. We proposed that the gender would moderate the direct and/or indirect pathways from parenting style to college student’s emotional distress. We conducted an exploratory study and did not make specific predictions about the patterns of the moderation effect.

For age, a great many studies have shown that parenting style has a life-long impact on the offspring (Zhong et al., 2016; Ernst et al., 2020). We therefore hypothesized that age would not moderate the direct or indirect correlations between parenting style and both depression and anxiety symptoms among college students.

SES is another important family factor which has great influence on the development of individuals. Many prior studies have shown that low level of SES is associated with elevated socioemotional and academic adjustment problems (Van Oort et al., 2011; Sun et al., 2018). Furthermore, SES was reported to be associated with parenting styles. For instance, parents with low levels of income and education are more likely to discipline the offspring in a punitive manner and ignore the offspring’s dependency and emotional needs (Hoff et al., 2002). In addition, research has also indicated that family SES is associated with the offspring’s personality development. For instance, the adults whose parents had more years of education were more emotionally stable, open, and extraverted (Sutin et al., 2017). Strickhouser and Sutin (2019) have also found that children with lower family SES tended to have lower sociability, higher reactivity, and lower persistence and these associations did not decrease over time. Finally, the stress-buffering model posits that the deleterious effect of stressful contexts on individual development will be smaller for those with many protective factors (Cohen and Wills, 1985). This is because protective factors can alleviate the adverse impacts of stressful contexts. Extrapolating this theory to parenting, high SES could alleviate the deleterious effects of negative parenting and thus the impact of negative parenting on Zhongyong thinking and emotional distress is weaker for college students with high SES. Building on these, we hypothesized that SES would moderate the direct and/or indirect pathways from parenting styles to emotional distress. Specifically, compared with high SES students, stronger direct and/or indirect pathways from parental rejection and overprotection to emotional distress would be found for low SES; for parental warmth and emotional distress, we conducted an exploratory study and did not make specific assumptions about the patterns of the moderation effect.

The purpose of the present study was to investigate how and under what condition parenting style could be associated with emotional distress among Chinese college students. Specifically, this study sought to expand the literature by specifying the mechanisms underlying and circumstances surrounding the association between parenting style and emotional distress by considering the Confucianism personality-Zhongyong thinking as a potential mediator and the gender, age and SES as potential moderators. On the basis of theories and prior research, we hypothesized that higher levels of parental rejection and overprotection as well as lower levels of parental warmth would correlate with more emotional distress (anxiety and depression) through lower Zhongyong thinking (H1: the mediating hypothesis). We also hypothesized that gender and SES would moderate the direct and/or indirect pathways from parenting style to emotional distress and that age did not have a moderating role (H2, H3, and H4: the moderating hypotheses).

## Materials and Methods

### Participants

The participants of the current study included college students who came from biological families with both parents. The stratified cluster sampling was used to recruit college students to participate in the present study. Four universities (Guangdong University of Technology, Guangdong University of Science and Trade, Southern Medical University, and South China Agricultural University) were chosen from Guangdong province in mainland China, and 100 classes were then chosen. Reasons for non-response included non-attendance of the survey class (97 students) and withdrawing before the questionnaire was completed (12 students). Of the 4081 questionnaires collected in this study, 109 surveys were excluded due to incompleteness and 29 surveys were excluded due to the unreasonable answers (for instance, the participant reported that his/her parent had high levels of overprotection and rejection at the same time; the participant got the lowest score in one of the dimension of Zhongyong thinking, while got the highest scores in other two dimensions), resulting in 3943 valid questionnaires (valid response rate was 96.62%). No statistically significant difference was found in demographic characteristics between the included and excluded cases. The age range of the valid sample was 18 to 26 years (Mean age = 21.32 years, SD age = 1.38 years). In this sample, 2433 (61.70%) were females, and 1510 (38.30%) were males; 3826 (97.03%) were of Han nationality, and 117 (2.97%) were minorities.

### Procedure

The procedure was approved by institutional review board (Number 2012ZGXM-0006) prior to beginning of the study. The investigators were trained to manage questionnaires under the same instructions and provided help or clarification if needed, thereby ensuring the effectiveness of data collection. Before taking part in this survey, all students had been told about the purpose of the present study and the voluntary nature of participation. Students who agreed to participate were guided to complete the questionnaire anonymously and confidentially in their classrooms. After completing the questionnaire, each participant received 10 RMB payments.

### Measures

#### Parenting Styles

Participants completed the Chinese version of the Short-Form of the Egna minnen av Barndoms uppfostran (One’s Memories of Upbringing) (s-EMBU-c) (Jiang et al., 2010) to assess individual’s own memories of perceived parental behaviors (Zhang et al., 2019). The s-EMBU-c is translated and modified from the English version of s-EMBU (Arrindell et al., 1999) and assesses perceptions of parental rejection (12 items), parent warmth (14 items), and parental overprotection (16 items) (Fu et al., 2015; Zhang et al., 2019). Each item is rated on a 4-point Likert scale (1 = No, never; 2 = Yes, but seldom; 3 = Yes, often; 4 = Yes, most of the time). Total scores range from 12 to 48 for parental rejection, 14 to 56 for parent emotional warmth, and 16 to 64 for parental overprotection, with higher scores demonstrating higher levels of parental behaviors. The revised Chinese version has good reliability and validity (Xu et al., 2017; Zhang et al., 2019). In this study, the Cronbach’s alpha coefficient was 0.86 for parental rejection, 0.91 for parent emotional warmth, and 0.82 for parental overprotection.

#### Zhongyong Thinking Style

Zhongyong thinking style was measured by the Chinese version of the Zhongyong thinking Style Scale (ZYTS) (Wu and Lin, 2005). It is a 13-item scale that measures three dimensions of Zhongyong thinking: multi-thinking (four items), holism (five items), and harmoniousness (four items). Participants are asked to evaluate their cognitive process in a hypothetical situation and rate items on a 5-point Likert scale (0 = strongly disagree, 4 = strongly agree). Total scores range from 0 to 16 for both multi-thinking and harmoniousness, and from 0 to 20 for holism ranges, with higher scores demonstrating higher Zhongyong thinking. The scale has shown acceptable reliability and validity (Wu and Lin, 2005; Yang et al., 2016; He et al., 2017). For instance, in the study of Wu and Lin (2005), the internal consistence coefficient of multi-thinking, holism, harmoniousness, and the total Zhongyong thinking was 0.62, 0.73, 0.79, and 0.87, respectively. In this study, the Cronbach’s alpha for the total scale was 0.89, and those for multi-thinking, holism, and harmoniousness were 0.61, 0.78, and 0.84, respectively.

#### Depressive Symptoms

The Chinese version of the Self-Rating Depression scale (SDS) (Zung, 1999) was used to assess current depressive symptoms. It is translated from the original English version of the SDS (Zung, 1967). There are 20 items on the Chinese version of the scale, either positive or negative, which the study subjects are required to grade on a scale of 1 (none or a little of the time) to 4 (most or all of the time). The total scores range from 20 to 80, with higher scores indicating greater depressive symptom severity. The SDS has been tested for validity and reliability and found to be acceptable for Chinese samples (Peng et al., 2013). In this study, the Cronbach’s alpha was 0.82.

#### Anxiety Symptoms

Anxiety was measured by Chinese version of the Self-Rating Anxiety scale (SAS) (Wang and Chi, 1984). It was translated and modified from the English version (Zung, 1971). The SAS contains 20 items that represent commonly found anxiety symptoms. Agreement with statements is assessed on a 4-point Likert-type scale ranging from 1 (none or a little of the time) to 4 (most or all of the time). Therefore, the total scores of the scale range from 20 to 80, with a higher score representing more anxiety symptoms. The SAS has demonstrated adequate validity and reliability for anxiety symptoms in Chinese samples (Xu and Wei, 2013). In this study, the Cronbach’s alpha was 0.75.

#### SES

SES factors included in present study comprised of maternal education, paternal education, and family economy. Participants reported the level of education that both their mother and father achieved from 1-5 (1 = less than elementary education or 6 years of schooling, 2 = junior middle school studies or 9 years of schooling, 3 = senior middle school studies or 12 years of schooling; 4 = bachelor’s degree or 16 years of schooling; 5 = more than master’s degree or 17 years of schooling. Participants were also asked to rate their family economy either as very poor, poor, fair, good, or very good, scored as 1 to 5. As some college students may not know the exact annual income of their family, this index was not recorded. We added up the answers to the above three questions to get the total score of SES. Then the total SES was recorded as a dichotomous variable (low and high SES groups) based on the median split.

### Statistical Analysis

Statistical analyses were performed using SPSS 20.0 for Windows and AMOS 7.0. Data were first screened for outliers and to assess linearity and normality. According to Kim (2013), an absolute skew value less than 2 can be considered to be within the typically acceptable range of normality. Descriptive statistics and Pearson correlation analyses were performed among the study variables. Then, *t*-tests were used to compare parenting style, depressive symptoms, anxiety symptoms, and Zhongyong thinking between genders, ages, and SES groups.

Structural equation modeling (SEM) with the AMOS software package was used to test the mediating effect hypotheses. In this analysis, Zhongyong thinking as a latent variable was assessed by multi-thinking, holism, and harmoniousness, and manifest variables included parental rejection, parent emotional warmth, parental overprotection, SDS, and SAS. The maximum likelihood estimation method was used. Following established recommendations (Wu, 2009), overall model fit was tested by considering several fit indexes. Models were compared based on χ^2^ tests and on other fit indexes: the Bentler comparative fit index (CFI), the Normed fit index (NFI), the incremental fit index (IFI), and the root mean square error of approximation (RMSEA). For CFI, IFI, and NFI, values greater than 0.90 represent a good model fit, and for RMSEA, values less than 0.05 indicate a good model fit. The ratio of χ^2^ to degrees of freedom (χ^2^/df) was also used, and values less than 5 indicate an ideal fit. In the mediation analysis, bootstrapping was used to obtain confidence intervals (CIs) based on 10,000 samples (Preacher and Hayes, 2008).

Then, we identified sociodemographic characteristics such as student gender, age, and SES as possible moderator variables of the above mediating model using multi-group analysis, a special case of SEM. Genders were recorded as female and males, and the age and SES variables were recorded into dichotomous variables by means of median splits. As was recommended by Wu (2013), the tests of sociodemographic characteristic differences in the SEM framework were as follows. First, the hypothesized structure was tested without constraining any parameter in both groups simultaneously (named unconstrained model or baseline model). If the baseline model was of adequate fit, we forced certain parameters (i.e., measurement weights, measurement residuals, and structural residuals) to be equal for both groups (named constrained model) and compared the constrained and baseline models. If the statistical fit of the constrained model revealed a significantly worse solution (the significance of the increase in χ^2^ values) than the unconstrained one, this suggests that at least one of the parameters is different across groups. If more than one model yielded adequate data-model fit, the final model was selected according to Δχ^2^, AIC, and ECVI indexes.

## Results

### Descriptive Statistics and Univariate Correlations of Study Variables

Descriptive statistics and correlations are displayed in Table 1. The skewness values were less than 2, indicating that the study variables were not substantially skewed. As expected, all variables were significantly correlated with each other. Specifically, college student’s parental rejection and over-protection negatively related to their Zhongyong thinking, while parental emotional warmth positively related to it. Zhongyong thinking was negatively correlated with depressive and anxiety symptoms. College student’s parental rejection and overprotection had significant and negative associations with depressive and anxiety symptoms, and parental emotional warmth had a positive association with them.

**TABLE 1:** Descriptive statistics and univariate correlations of study variables.

Variables	Skewness	*M*	*SD*	Correlations (*r*)								
				1	2	3	4	5	6	7	8	9
1. Parent rejection	1.75	15.98	4.62	1								
2. Parent emotional warmth	–0.40	41.96	7.91	−0.349**	1							
3. Parent over-protection	0.47	31.85	6.86	0.485**	−0.112**	1						
4. Multi-thinking	–0.79	11.51	2.51	−0.210**	0.252**	−0.105**	1					
5. Holism	–0.84	15.01	2.84	−0.200**	0.257**	−0.090**	0.634**	1				
6. Harmoniousness	–0.74	12.36	2.41	−0.162**	0.281**	−0.061**	0.626**	0.694**	1			
7. Zhongyong Thinking	–0.96	38.89	6.80	−0.219**	0.299**	−0.098**	0.856**	0.897**	0.875**	1		
8. SDS	0.47	34.59	7.43	0.294**	−0.314**	0.201**	−0.306**	−0.298**	−0.293**	−0.341**	1	
9. SAS	0.87	32.10	6.33	0.297**	−0.209**	0.234**	−0.214**	−0.203**	−0.207**	−0.237**	0.695**	1

SDS = Self-Rating Depression Scale; SAS = Self-Rating Anxiety Scale; **p < 0.01.

### Comparisons of Study Variables

Table 2 shows the *t*-test results regarding the student-reported parenting styles, Zhongyong thinking, and emotional distress (depression and anxiety symptoms) under different demographic variables. The results revealed that the younger group scored higher on parental rejection, parental overprotection, depressive symptoms, and anxiety symptoms than the older group, while the older group scored higher on multi-thinking. Significant gender effects were found for parenting styles and Zhongyong thinking (Table 2). Specifically, males had higher scores than females on parental rejection and overprotection, while females had higher scores than males on parent emotional warmth as well as Zhongyong thinking and its dimensions of holism and harmoniousness. In addition, high SES students scored higher on parent emotional warmth as well as Zhongyong thinking and its three dimensions, while low SES students scored higher on anxiety and depressive symptoms. Cohen’s *d* effect size values for *t* tests indicated that all these differences were of small effect (Cohen, 1992).

**TABLE 2:** Comparisons of study variables between younger and older students, female and male students, and between Low SES and High SES students.

	18–21 years		22–26 years		*p*	*d*	Female		Male		*p*	*d*	Low SES		High SES		*p*	*d*
	*M*	*SD*	*M*	*SD*			*M*	*SD*	*M*	*SD*			*M*	*SD*	*M*	*SD*		
Parent rejection	16.28	4.73	15.53	4.40	< 0.001	0.163	15.62	4.26	16.55	5.08	< 0.001	–0.203	16.02	4.55	15.93	4.68	0.564	0.020
Parent emotional warmth	41.90	7.72	42.04	8.16	0.577	–0.018	42.24	7.87	41.50	7.95	0.004	0.094	40.59	7.86	43.41	7.69	< 0.001	–0.363
Parent over-protection	32.27	6.76	31.23	6.94	< 0.001	0.152	31.43	6.99	32.53	6.57	< 0.001	–0.161	31.71	6.68	32.00	7.03	0.183	–0.042
Multi-thinking	11.40	2.51	11.67	2.51	0.001	–0.108	11.50	2.34	11.52	2.77	0.826	–0.008	11.37	2.45	11.66	2.57	< 0.001	–0.116
Holism	15.00	2.79	15.03	2.90	0.719	–0.011	15.15	2.62	14.79	3.15	< 0.001	0.127	14.88	2.79	15.16	2.89	0.002	–0.099
Harmoniousness	12.34	2.37	12.38	2.45	0.623	–0.017	12.47	2.21	12.17	2.67	< 0.001	0.125	12.24	2.39	12.48	2.41	0.002	–0.100
Zhongyong Thinking Style	38.75	6.68	39.10	6.99	0.118	–0.051	39.13	6.20	38.50	7.67	0.004	0.093	38.56	6.64	39.31	6.95	< 0.001	–0.110
SDS	34.84	7.35	34.22	7.51	0.010	0.084	34.55	7.26	34.64	7.69	0.712	–0.012	35.07	7.35	34.07	7.47	< 0.001	0.135
SAS	32.44	6.36	31.59	6.23	< 0.001	0.135	32.21	6.32	31.92	6.32	0.168	0.046	32.32	6.26	31.86	6.38	0.023	0.073

*n = 2343 for the 18–21 years, n = 1600 for the 22–26 years; n = 2433 for female, n = 1510 for male; n = 2027 for low SES, n = 1916 for high SES. SES = socioeconomic statuses; SDS = Self-Rating Depression Scale; SAS = Self-Rating Anxiety Scale. d = Cohen’s d.*

### The Mediating Effect of Zhongyong Thinking

**FIGURE 1:**
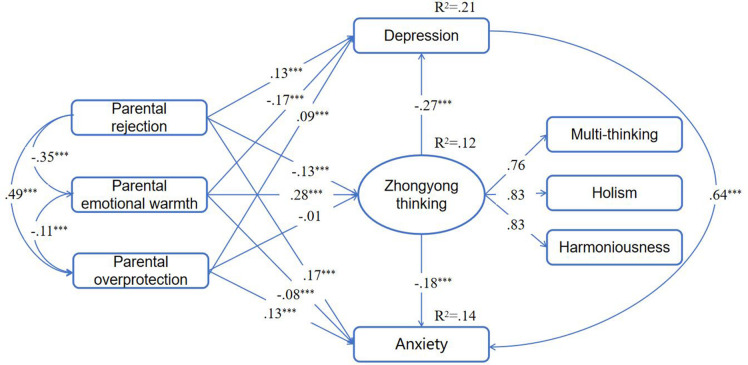
College students’ Zhongyong thinking as mediator between parenting styles and both depression and anxiety. Standardized path coefficients are presented in the model. ***p < 0.001.

The overall fit indexes revealed a good fit between the model and the data (χ^2^/df = 4.580; RMSEA = 0.030, 90% CI = 0.022, 0.039; CFI = 0.997, NFI = 0.996, IFI = 0.997). As shown in Figure 1, factor loadings were greater than 0.76 for Zhongyong thinking. These findings showed that the parceling method was efficient.

The results indicated that parental rejection and parental warmth had a significant negative and positive association with Zhongyong thinking, respectively, (β = −0.13, *p* < 0.001; β = 0.28, *p* < 0.001). However, the direct path from parental overprotection to Zhongyong thinking was not significant (β = −0.01, *p* = 0.711). College student’s Zhongyong thinking was negatively linked with both depression and anxiety symptoms (β = −0.27, *p* < 0.001; β = −0.18, *p* < 0.001). The direct path from parental rejection, overprotection, and warmth to depression (β = 0.13, *p* < 0.001; β = −0.17, *p* < 0.001; β = 0.09, *p* < 0.001) and anxiety symptoms were all significant (β = 0.17, *p* < 0.001; β = −0.08, *p* < 0.001; β = 0.13, *p* < 0.001). Furthermore, parental rejection and warmth were significantly and indirectly related to depression (standardized indirect effect = 0.04, 95% BCIs:0.02, 0.05; standardized indirect effect = −0.08, 95% BCIs: −0.09, −0.06) and anxiety symptoms (standardized indirect effect = 0.02, 95% BCIs:0.02, 0.03; standardized indirect effect = −0.05, 95% BCIs: −0.06, −0.04).

### The Moderating Effect of Age, Gender, and/or SES: Multi-Group Moderation Analysis

We also addressed the potential moderating effects of age, gender, and SES. To investigate these effects, we tested whether the relationships between parenting styles and the dependent variables found for the whole sample also held for different subgroups.

For age, the unconstrained baseline model and all the constrained models (measurement weights, structural covariances, and measurement residuals) fit the data well (Table 3). The results revealed a non-significant χ^2^ difference for the unconstrained baseline model and the measurement weights model. However, the differences in the χ^2^ values between the unconstrained baseline model and both the structural covariances model and the measurement residuals model were significant, indicating that at least one of the parameters was different across ages. Although all four models yielded adequate data-model fit, we selected the measurement weights model as the final model according to χ^2^, AIC, and ECVI indexes. Then, we compared path coefficients for students aged 18–21 years and those aged 22–26 years one by one and found that there were no significant differences between all the path coefficients for the two groups. Results of these indicated that the meditating model demonstrated invariance across ages.

**TABLE 3:** Goodness of fit indices for model comparisons in moderation analysis of age, gender, and socioeconomic status on the mediation model.

Model	χ ^2^	*df*	χ ^2^/df	Δ χ ^2^	Δ df	*p*-Value for Δ χ ^2^	RMSEA (90% CI)	CFI	NFI	IFI	AIC	ECVI
Threshold for acceptable fit			<5			≥0.001 (significant level)	<0.05	≥0.90	≥0.90	≥0.90		
**Age**												
Unconstrained	55.717	20	2.786				0.021 (0.015–0.028)	0.997	0.995	0.997	159.717	0.041
Measurement weights	56.928	24	2.372	1.211	4	0.876	0.019 (0.012–0.025)	0.997	0.995	0.997	152.928	0.039
Structural covariances	92.827	39	2.380	37.111	19	0.008	0.019 (0.014–0.024)	0.995	0.991	0.995	158.827	0.040
Measurement residuals	115.710	46	2.515	59.993	26	< 0.001	0.020 (0.015–0.024)	0.994	0.991	0.994	167.710	0.043
**Gender**												
Unconstrained	60.128	20	3.006				0.022 (0.016–0.029)	0.996	0.994	0.996	164.128	0.042
Measurement weights	76.843	24	3.202	16.716	4	0.002	0.024 (0.018–0.030)	0.995	0.993	0.995	172.843	0.044
Structural covariances	205.066	39	5.258	144.939	19	< 0.001	0.033 (0.028–0.037)	0.984	0.981	0.984	271.066	0.069
Measurement residuals	298.891	46	6.498	238.764	26	< 0.001	0.037 (0.033–0.041)	0.976	0.972	0.976	350.891	0.089
**Socioeconomic status**												
Unconstrained	55.013	20	2.751				0.021 (0.014–0.028)	0.997	0.995	0.997	159.013	0.040
Measurement weights	59.237	24	2.468	4.223	4	0.377	0.019 (0.013–0.026)	0.997	0.994	0.997	155.237	0.039
Structural covariances	103.570	39	2.656	48.557	19	< 0.001	0.020 (0.016–0.025)	0.994	0.990	0.994	169.570	0.043
Measurement residuals	108.863	46	2.367	53.850	26	0.001	0.019 (0.014–0.023)	0.994	0.990	0.994	160.863	0.041

*χ^2^ = chi-square; df = degree of freedom; χ^2^/df = The chi-squared/freedom ratio; RMSEA = Root mean square error of approximation; CFI = Comparative fit index; NFI = Normed fit index; IFI = Incremental fit index; AIC = Akaike information criteria; ECVI = Expected cross-validation index. The chi-square deference (Δχ^2^) tests are compared with the unconstrained model (baseline model).*

As for gender, although the values of χ^2^/df for the structural covariances model and the measurement residuals model were greater than 5, the other indexes were good, suggesting that the hypothesized models were acceptable. Meanwhile, the other two models fit the data well. Compared with the unconstrained model, however, the differences in the χ^2^ values were significant, indicating that the data model fit significantly changed compared with that of the unconstrained model. Considering χ^2^, AIC, and ECVI indexes, we selected the unconstrained model as the final model. In further analysis, we found that the positive link between parental warmth and Zhongyong thinking was stronger for males than females (for males, β = 0.31, *p* < 0.001; for females, β = 0.26, *p* < 0.001; *t* = 3.33, *p* < 0.001).

Concerning the proposed moderator effect by SES, all four models fit the data well. There was no significant difference between the unconstrained baseline model and the measurement weights model. However, the differences in the χ^2^ values between the unconstrained baseline model and both the structural covariances model and the measurement residuals model were significant. We selected the measurement weights model as the final model according to the same criteria. The results showed that the relationship between parental warmth and Zhongyong thinking was stronger (*t* = 2.17, *p* < 0.05) for high SES students (β = 0.30, *p* < 0.001) than for low SES ones (β = 0.25, *p* < 0.001). Furthermore, the relationship between parental overprotection and Zhongyong thinking was not significant for high SES students (β = 0.04, *p* = 0.132) but significant for low ones (β = −0.06, *p* = 0.030), and the difference in the pathways was significant (*t* = 2.61, *p* < 0.01).

## Discussion

Consistent with the hypothesis, the current study showed that parenting style was mildly associated with emotional distress among Chinese college students and that Zhongyong thinking mediated the relationships between them. Specifically, when students perceived more warmth and less rejection, they were more likely to develop a high level of Zhongyong thinking, which was negatively associated with both depressive and anxiety symptoms. Also in line with the assumptions, the gender and SES moderated the mediating model. Specifically, the positive association between parental warmth and Zhongyong thinking was stronger for males and for high SES students; the negative link between parental overprotection and Zhongyong thinking was stronger for low SES students. Expectedly, the mediating model results were similar for both younger and older students. In view of college student’s emotional distress being on the rise (Auerbach et al., 2018), our findings here extend the current literature, indicating that the Confucianism personality-Zhongyong thinking is an important mediator for the relationship between parenting style and emotion among Chinese college students.

Congruent with previous research (Gao et al., 2012; Zhong et al., 2016; Cui, 2017), we found a positive association of parental rejection and overprotection with depression and anxiety symptoms and a negative association of parental warmth with depression and anxiety among adult college students. It is also consistent with a meta-analytic review of the link between parenting styles and both depressive and anxiety symptoms among adolescents that concluded that less parental warmth and over-involvement were linked with increased risks of depression and anxiety (Yap et al., 2014). These suggests that when parents had a warm attitude toward their offspring and expressed more care and support toward them, the offspring were less likely to develop depressive and anxiety symptoms. However, if individuals perceived high levels of parental rejection or overprotection, they were more likely to become depressed and anxious. These might be useful for parents to rethink about their parenting practices, for instance, to improve or adjust their parenting behaviors, so as to decrease the likelihood of emotional distress in their offspring.

This study further demonstrated that Zhongyong thinking was negatively and moderately correlated with depressive symptoms, and negatively and mildly correlated with anxiety symptoms. This result was consistent with previous findings (Zhan et al., 2013; Yang et al., 2016). Zhongyong thinking is therefore related to emotional distress of Chinese college students. The possible reasons for this might be as follows. Firstly, as Zhongyong stresses the importance of maintaining interpersonal harmony (Wu and Lin, 2005), it facilitates more social support (Chuang, 2005) that acts as a critical protective factor for emotion in Chinese culture (Moak and Agrawal, 2009). Meanwhile, Zhongyong thinking emphasizes accepting the coexistence of negative and positive emotions and the emotional complexity from hindrance-related stress, thus facilitating emotion regulation and relieving emotional distress (Spencer-Rodgers et al., 2010). Furthermore, Zhongyong thinking encourages people to view current suffering and distress from multiple perspectives (Masuda and Nisbett, 2001), which is beneficial for individuals in emotional distress to converse or alleviate their painful experience (Yang et al., 2016). Therefore, individuals with high Zhongyong thinking might be less inclined to depression and anxiety symptoms.

Taken together, we found significant mediating effects of Zhongyong thinking on the associations of parental rejection with emotional distress as well as of parental warmth with emotional distress. Specifically, college students who perceived less rejection and more emotional warmth were more likely to develop high levels of Zhongyong thinking, which consequently might protect individuals from emotional distress. Conversely, if parents communicate with their children in a refusing way, it might impede the development of Zhongyong thinking, leading to emotional distress. These results were also in line with previous reports which implied that parenting styles might influence adult’s emotional distress indirectly through their associations with personality traits, such as self-control (Hong and Cui, 2020) and resilience (Zhong et al., 2016). The mediating effect of Zhongyong thinking could be partially accounted for as follows. Neither in the extreme of laxity nor in the extreme of excessive punishment, parent emotional warmth is in the middle of the continuum of parenting behaviors, which is similar to the idea of Zhongyong thinking. Meanwhile, parents who show more warmth for their offspring are usually the ones who had better emotion regulation strategies, which is a prominent feature of the high Zhongyong thinking individuals. Therefore, college students whose parents are emotionally warm might tend to have more opportunity to model their parents and develop Zhongyong thinking, which is associated with decreased emotional distress. Furthermore, to the extent that parent-child interactions are characterized by emotional warmth, a secure attachment develops (Jia et al., 2019) that leads to an adaptive personality (Ulu and Tezer, 2010; Nazzaro et al., 2017), which is an effective way to diminish emotional distress. On the contrary, parental rejection runs counter to the idea of Zhongyong thinking and might cause disharmony between parents and their offspring. The parent-child disharmony may hinder the college students from forming culturally adapted personality-Zhongyong thinking.

Notably, in the whole sample, although the indirect effects of parental overprotection on depression and anxiety were not statistically significant, its direct effects were. Parental overprotection, therefore, is still relevant for college student’s elevated emotional distress. This might be that parental overprotection hinders the development of other personality of college students, such as personal autonomy and competence, which are associated with high emotional distress (Schiffrin et al., 2014; Kouros et al., 2017).

Most importantly, this study was the first, to our knowledge, to explore whether personal characteristic (age and gender) and environmental characteristic (SES) moderated the mediated effect of parenting style on emotional distress through Zhongyong thinking. The present study showed that for college students who perceived the same level of parental warmth, male students were expected to develop more Zhongyong thinking than females. The gender intensification hypothesis might partly account for this. According to this hypothesis, parent’s emphasis on traditional gender roles and offspring’s awareness of gender-related roles are both enhanced beginning in adolescence (Hill and Lynch, 1983). Specifically, males are guided to be independent, autonomous, and assertive, while females are directed to be obedient. For example, it was reported that males received more autonomy-support than females (Bumpus et al., 2001; Lanza et al., 2012). It is reasonable to infer that the autonomy-support and independence formed by males is beneficial to their development of multiple and holistic thinking. When perceived the same degree of parental warmth, males therefore might develop more Zhongyong thinking than females.

The present study indicated that SES moderated the paths from parental warmth and overprotection to Zhongyong thinking. Specifically, the positive association of parental warmth and Zhongyong thinking was stronger for high SES college students, while the negative association of parental overprotection and Zhongyong thinking was stronger for low SES ones. These results demonstrated that low SES seemed to be link with enhanced negative association of parental overprotection-Zhongyong thinking and decreased positive association of parental warmth-Zhongyong thinking. This is congruent with the stress-buffering model (Cohen and Wills, 1985). Compared with higher SES college students, college students with lower SES typically obtain less emotional and material support (such as rarely being listened to talk about daily life, getting less valuable information and advice, and receiving less frequent technology help and material support) (Hoff et al., 2002; Fingerman et al., 2015), which might not be enough to buffer the negative associations of parental rejection and emotional distress among them. Findings of these suggest that future research into the relationship between parenting style, Zhongyong thinking and emotional distress in low SES college students is urgently required, especially on the reduction of the negative association of parental overprotection-Zhongyong thinking and the elevation of the positive association of parental warmth-Zhongyong thinking.

However, we did not find the moderating effect of age on the association of parental rejection or warmth with emotional distress. In other words, the relationships of parental rejection and warmth to emotional distress through Zhongyong thinking did not vary with age. One possible explanation is that Zhongyong thinking might be closely related to the emotional distress associated with high parental rejection and low warmth. This once again expands the previous literature and supports the important and long-term impact of parenting style on the offspring (Tani et al., 2018; Ernst et al., 2020). It should be noted that the age range of the participants in this study is small (from 18 to 26 years old). There the moderating role of age needs to be examined among participants with a larger age range in future.

## Limitations and Conclusion

Some limitations of the current study should be noted. Firstly, the evaluation of parenting style was based on college student’s self-report, which might differ from parental reports. It would be beneficial for future studies to also include parental reports. Secondly, as all the college students in present study came from biological families with both parents, the findings of present study may not be generalized to these college students in non-biological families. Thirdly, the data in this study were collected in 2012 and the internal consistency for the multi-thinking dimension of Zhongyong thinking was relatively low. New data are needed to repeat the results in present study. Then, in addition to common paths through which parents impacted college student’s Zhongyong thinking and emotional distress, there might also be separate paths by which the father/mother may had a unique impact on them. Therefore, future studies should separately assess these pathways to obtain a more complete picture on the relationships among paternal/maternal parenting style, Zhongyong thinking, and emotional distress. Furthermore, similar to previous cross-sectional studies, our results failed to provide a temporal sequence and definitive etiological conclusion. Longitudinal studies are needed to assess potential causal relationships from a developmental perspective. Finally, the association between parenting style and college student’s emotional distress in this study was relatively small. However, in view of the fact that parents have a prolong effect on college student’s personality and mental health, the small association observed may have a momentous practical influence on college students over time.

Despite these limitations, the present study adds to the literature for the direct effect of parenting style on emotional distress among Chinese college students. Furthermore, this study is the first to find that parenting warmth and rejection were indirectly correlated with emotional distress through the Confuciaism personality–Zhongyong thinking. That is, Zhongyong thinking can serve as a beneficial personality feature for understanding the relationship between parenting style and emotional distress among college students. It is also worth mentioning that the mediating effect of Zhongyong thinking for parenting style varies with gender and SES. Based on these, the present study highlights the consideration of both personal and environmental factors to prevent emotional distress for Chinese college students. In addition, findings of current study not only advance the understanding of how Zhongyong thinking affects the emotion but also enrich the Zhongyong thinking research in the field of family context. Furthermore, future parenting programs to assist Chinese parents should encourage parental emotional warmth and decrease parental rejection to facilitate the development of Zhongyong thinking and eliminate emotional distress of their offspring. Finally, the findings are useful for clinicians or psychotherapists working with Chinese young adult’s emotional distress. Specifically, although college students are already adults, the parent and family context continue to be important influencing factors for them; and Zhongyong thinking is worth training in the therapeutic settings for these emerging adults.

## Data Availability Statement

The datasets used and/or analyzed during the current study are available from the corresponding author on reasonable request.

## Ethics Statement

The studies involving human participants were reviewed and approved by Southern Medical University Ethics Committee. The patients/participants provided their written informed consent to participate in this study.

## Author Contributions

XZh designed the study and wrote the protocol. YH and XY assisted with the survey and managed the literature searches. YH performed the statistical analysis and wrote the first draft of the manuscript. RX made substantial contributions to the analysis and interpretation and drafted the revision. FP, XY, and YC contributed to the conception of the study, results interpretation, and edited the final manuscript. SZ and XZe edited the final manuscript. All authors contributed, read, and approved the final manuscript.

## Conflict of Interest

The authors declare that the research was conducted in the absence of any commercial or financial relationships that could be construed as a potential conflict of interest.
